# Measurement of the Anisotropic Dynamic Elastic Constants of Additive Manufactured and Wrought Ti6Al4V Alloys

**DOI:** 10.3390/ma15020638

**Published:** 2022-01-15

**Authors:** Ofer Tevet, David Svetlizky, David Harel, Zahava Barkay, Dolev Geva, Noam Eliaz

**Affiliations:** 1 Department of Materials Science and Engineering, Tel Aviv University, Ramat Aviv, Tel Aviv 69978, Israel; tevet.ofer@gmail.com (O.T.); dsvetlizky@gmail.com (D.S.); dharel@tauex.tau.ac.il (D.H.); 2 Materials Department, Nuclear Research Center Negev (NRCN), Beer Sheva 84190, Israel; 3 The Wolfson Applied Materials Research Centre, Tel Aviv University, Ramat Aviv, Tel Aviv 69978, Israel; barkay@tauex.tau.ac.il; 4 Israel Ministry of Defense, Hakirya, Tel Aviv 61909, Israel; dolev24@yahoo.com

**Keywords:** Ti6Al4V, additive manufacturing (AM), directed energy deposition (DED), electron beam melting (EBM), selective laser melting (SLM), wrought alloy, pulse-echo ultrasonic technique, dynamic elastic constants, Young’s modulus, shear modulus, Poisson’s ratio

## Abstract

Additively manufactured (AM) materials and hot rolled materials are typically orthotropic, and exhibit anisotropic elastic properties. This paper elucidates the anisotropic elastic properties (Young’s modulus, shear modulus, and Poisson’s ratio) of Ti6Al4V alloy in four different conditions: three AM (by selective laser melting, SLM, electron beam melting, EBM, and directed energy deposition, DED, processes) and one wrought alloy (for comparison). A specially designed polygon sample allowed measurement of 12 sound wave velocities (SWVs), employing the dynamic pulse-echo ultrasonic technique. In conjunction with the measured density values, these SWVs enabled deriving of the tensor of elastic constants (*C*_ij_) and the three-dimensional (3D) Young’s moduli maps. Electron backscatter diffraction (EBSD) and micro-computed tomography (μCT) were employed to characterize the grain size and orientation as well as porosity and other defects which could explain the difference in the measured elastic constants of the four materials. All three types of AM materials showed only minor anisotropy. The wrought (hot rolled) alloy exhibited the highest density, virtually pore-free μCT images, and the highest ultrasonic anisotropy and polarity behavior. EBSD analysis revealed that a thin β-phase layer that formed along the elongated grain boundaries caused the ultrasonic polarity behavior. The finding that the elastic properties depend on the manufacturing process and on the angle relative to either the rolling direction or the AM build direction should be taken into account in the design of products. The data reported herein is valuable for materials selection and finite element analyses in mechanical design. The pulse-echo measurement procedure employed in this study may be further adapted and used for quality control of AM materials and parts.

## 1. Introduction

Ti6Al4V alloy was developed in the 1950s for the aerospace industry, which is still its largest consumer [1,2]. However, thanks to its unique combination of properties, such as high strength, high fracture toughness, excellent corrosion resistance, superior biocompatibility, and low density, Ti6Al4V has also become common in the energy, chemical, marine, automobile, and biomedical industries [1,3]. Due to the significant advantages of additive manufacturing (AM) over traditional Ti6Al4V manufacturing processes, for example, the ability to form near-net-shape parts and complex geometries, a relatively short lead time, design flexibility, and minimal material waste, the interest in AM of Ti6Al4V has rapidly increased [1,4,5,6,7,8]. Despite the abovementioned advantages of metal AM in general, and AM of Ti6Al4V specifically, the ability to fabricate fully dense, defect-free parts with homogeneous microstructure, good surface finish, and good mechanical properties are still considered to be a challenge [9,10,11]. 

Metal-based AM technologies are typically based on a layer-by-layer deposition approach and are classified according to the type of feedstock used (powder vs. wire), the energy source (e.g., laser vs. electron beam), and the methodology of printing (e.g., direct deposition vs. powder bed). The two major metal AM processes are powder bed fusion (PBF) and directed energy deposition (DED). The former can further be divided into selective laser melting (SLM), electron beam melting (EBM), etc. 

Anisotropic materials have direction-dependent properties. Fully anisotropic materials have no planes of symmetry and are characterized by 21 elastic constants. A subset of anisotropic materials are orthotropic materials, which have two orthogonal planes of symmetry and whose properties are independent of direction within each plane. Such materials require nine independent elastic constants in their constitutive matrices. AM materials and hot rolled materials are typically considered to be orthotropic due to their grain structure, which is affected by the build direction and rolling direction, respectively. There is a direct relationship between the processing parameters, microstructure, and properties of AM materials. Among others, the energy source scan rate and power, deposition atmosphere, feedstock quality, and powder mass flow rate (PMFR, for DED) control the evolved thermal history during part fabrication, and thus may affect the resulting microstructure evolution, defects, and anisotropy of the microstructure and mechanical properties [4,9]. 

Many studies have reported anisotropic properties of AM Ti6Al4V [1,12,13,14,15,16,17,18,19,20,21,22,23,24,25]. Wang et al. [14] reported that the heterogeneous nucleation sites in the melt pool and the associated grain morphology of DED titanium alloy are highly dominated by the PMFR. By controlling the PMFR one can adjust the resulting grain morphology between near-equiaxed, columnar, and mixed grain morphology. In another study, Wolff et al. [12] reported anisotropy of the mechanical properties of DED Ti6Al4V. It was shown that among the three studied (100), (010), and (001) orientations, the (010) samples showed the highest residual stresses due to the thermal history during processing as well as the highest degree of anisotropy in mechanical properties. It was shown that samples closer to the center of the cubic deposit were characterized by higher Young’s modulus, lower elongation, and higher yield stress. Furthermore, the microstructural evolution and anisotropy are highly affected by the scanning strategy during AM processing [10]. The anisotropic properties of Ti6Al4V alloy fabricated using EBM were also studied by De Formanoir et al. [20] who reported that the build direction has a direct effect on the mechanical properties. It was shown that samples built vertically exhibited reduced yield strength compared to horizontally built samples. This phenomenon was attributed to anisotropic texture properties resulting in anisotropy of mechanical properties. The anisotropy in microstructure and mechanical properties is also affected by the grain size and morphology of AM parts. The thermal history and cooling rates control the grain size; the latter is finer at higher melt pool cooling rates [10]. Columnar grains are typically coarser than equiaxed grains, and yield anisotropic mechanical properties [10]. Yu et al. [22] concluded that the aspect ratio of the columnar grains in Ti6Al4V fabricated using SLM affects the anisotropy in the mechanical properties (microhardness and tensile strength) more than the crystal texture and symmetry. Among various developments in recent years, minor additions of some alloying elements and microstructure refinement have allowed development of AM Ti-based alloys with reduced microstructural and mechanical anisotropy [26,27,28].

The microstructure and level of porosity, *P*, also affect the sound wave velocity (SWV) and the elastic moduli. The level of porosity (volume fraction) can be deduced from the measured and theoretical densities:

(1) $$P=\frac{\rho_{\;t}-\rho }{\rho_{\;t}}$$

where *ρ* is the measured (bulk) density and *ρ* *_t_* is the theoretical density. Various models have been suggested to express the change in elastic moduli as a function of porosity [29,30,31], the most common ones are represented by Equations (2)–(6):

(2) $$\mathrm{Spriggs}\;C=C_{0}\exp\left(-bP\right)$$

(3) $$\mathrm{Wang}\;C=C_{0}\exp\left(-bP-cP^{2}\right)$$

(4) $$A\;\mathrm{linear}\;\mathrm{relation}\;C=C_{0}\exp\left(1-hP\right)$$

(5) $$\mathrm{Hasselman}\;C=C_{0}\exp\left[1+\frac{AP}{1-(1+A)P}\right]$$

(6) $$\mathrm{Phani}\;C=C_{0}\exp{\left(1-aP\right)}^{n}$$

where *C* and *C*_0_ are the elastic moduli of the porous and nonporous materials, respectively, *P* is the volume fraction of porosity, *a*, *b*, *c*, *h*, and *n* are empirical (material) constants, and *A* is a parameter determined statistically from the experimental data. 

The porosity dependence of the longitudinal (pressure) and shear (transverse) sound wave velocities (*V_l_* and *V*_t_, respectively) can be determined from the porosity dependence of the elastic moduli. These are also influenced by the pore shape [32] and particle/agglomerate sizes [33]. Shear waves oscillate perpendicular to the direction of propagation; they are relatively weak compared to longitudinal waves and can only travel in solids. Sol et al. [34] revealed the anisotropic nature of the elastic moduli of AM AlSi10Mg by focusing on the angle dependence of *V*_t_. A similar observation was recently reported [25] for Ti6Al4V prepared by laser PBF.

The dynamic pulse-echo ultrasonic technique is a nondestructive, efficient, and fast method for the measurement of the elastic properties of materials [34,35,36]. Some advantages of this technique over traditional destructive static methods were reported, for example: higher accuracy, sensitivity, and repeatability, especially when using small samples [35,37]. The pulse-echo technique has recently been utilized to determine the elastic constants of DED Al5083 using the time-of-flight (TOF) sound velocity method [37]. This technique was also utilized to investigate the degree of anisotropy of the elastic properties of SLM AlSi10Mg around the build direction [34].

To the best of our knowledge, the use of the dynamic pulse-echo ultrasonic technique to compare between the anisotropic elastic constants of an alloy prepared by different AM processes has not been reported before. This paper elucidates the anisotropic elastic properties (Young’s modulus, shear modulus, and Poisson’s ratio) of four Ti6Al4V alloys: three AM (by SLM, EBM, and DED) and one wrought alloy (for comparison). A specially designed polygon sample allowed measurement of 12 SWVs, which, together with the measured density, enabled deriving of the tensor of elastic constants and the three-dimensional (3D) Young’s moduli maps. Electron backscatter diffraction (EBSD) and micro-computed tomography (μCT) were employed to characterize the grain size and orientation as well as porosity and other defects which could explain the difference in the measured elastic constants of the four materials. The data reported herein is valuable for materials selection and finite element analyses in mechanical design. The pulse-echo measurement procedure employed in this study may be further adapted and used for quality control of AM materials and parts. 

## 2. Materials and Methods

### 2.1. Alloy Processing, Sample Preparation, and Dynamic Pulse-Echo Ultrasonic Testing

In this study, four bulk Ti6Al4V alloys prepared by four different manufacturing processes were studied:

(1) A parallelogram sample, 30 mm × 30 mm× 25 mm in dimensions, was machined from a rod manufactured by EBM at the AM Center of Rotem Industries Ltd. (Mishor Yamin, Israel) using an Arcam Q20 Plus EBM machine (Arcam AB, Gothenburg, Sweden) and a Ti6Al4V Grade 5 spherical powder with a size distribution of 45–106 μm [38,39,40]. Rod specimens 11 mm in diameter were orientated on the XY plane of the tray. Printing parameters were set to accelerating voltage of 60 kV, beam current of 28 mA, speed function of 32 (∼2400 mm/s base beam speed), and layer thickness of 90 μm. The temperature was maintained in the range of 750–850 °C. A chamber pressure of 4 × 10^−3^ mbar was regulated utilizing a helium leak valve [40]. The chemical composition (wt.%) of the as-printed alloy was 88.5 Ti, 7.7 Al, 3.8 V, 0.1352 O, 0.0066 C, 0.0052 N, and 0.0036 H [40].
(2) A parallelogram sample, 16 mm × 26 mm × 12 mm in dimensions, was machined from a cut piece of a fitting DED with a LENS MR-7 (Omega) system by Optomec, Inc. (Albuquerque, NM, USA) [7]. The fitting was printed using a Ti6Al4V Grade 5 spherical powder with a particle size range of 44–149 µm. Deposition was carried out using a standard head, laser power of 450 W, PMFR of 3.78 g/min, travel speed of 63.5 cm/min, and layer thickness of 381 μm. The travel speed was the same for both contour and hatch. The deposition strategy was 0, 90, 180, and 270 degrees sequentially per layer with a hatch spacing of 0.508 mm [7]. The chemical composition of this alloy (wt.%) was 89.37 Ti, 6.28 Al, 3.74 V, 0.395 Fe, 0.07 Mo, 0.05 Nb, 0.045 Cr, 0.024 Ni, 0.019 Si, and 0.009 C (the O, N, and H concentrations were not measured) [7].
(3) SLM disc sample, 77 mm in diameter and 12 mm in height, was AM using EOS M290 system (EOS GmbH, Freiburg, Germany) at the Israel Institute of Metals, Technion (Haifa, Israel) [41]. The printing parameters were: Ti6Al4V Grade 23 (ELI) powder, layer thickness of 60 μm, laser power of 340 W, laser beam scan speed of 1.25 m/s, laser beam focus diameter of 70 μm, hatch spacing of 40 μm, laser beam scanning strategy of a rotation of 67° in the direction of the laser beam path for each new layer, and gas flow rate of 0.6 L/min. The sample did not undergo any stress-relieving heat treatment after printing.
(4) A wrought Ti6Al4V Grade 23 rod, 25.4 mm in diameter, was produced by Dynamet, Inc. (Washington, PA, USA) by hot rolling [38,39]. The chemical composition of this alloy (wt.%) was 90.485 Ti, 5.558 Ti, 3.674 V, 0.232 Fe, 0.049 Mo, 0.022 Cr, 0.009 Mn, 0.0069 C, 0.1296 O, 0.0200 H, and 0.0086 N [38].

There are two major approaches for obtaining the elastic constants of orthotropic materials using the dynamic pulse-echo ultrasonic technique. The first approach is called the ultrasonic goniometry immersion technique [42] and is used mainly for the elastic characterization of flat composite samples. In this technique, the flat sample is rotated in a liquid bath, and the acoustic transducers are stationary. The second approach is sometimes referred to as the polygon method [43,44]. In this approach, acoustic transducers are placed on twelve sample wedges. With this geometry, 12 sound velocities can be measured (based on the TOF and the propagation distance): three longitudinal velocities, *V*_l,ii_ (i = 1–3), in three directions; six shear velocities in all three directions and in two polarities, *V*_t,ij_ (i = 1–3, j = 1–3, i ≠ j); and three shear velocities in the diagonal directions, *V*_si_ (i = 1–3). 

Table 1 summarizes the velocity notations, sound wave type, direction of propagation, and the polarity of the shear waves. The right column provides a visual description of the sound wave propagating in the sample. Dissimilarity between two shear SWVs measured on the same wedge (e.g., *V*_12_ vs. *V*_13_) would indicate an ultrasonic anisotropic behavior. Measuring the shear SVWs at several angles can elucidate the orthotropic nature of the sample.

**Figure 1:**
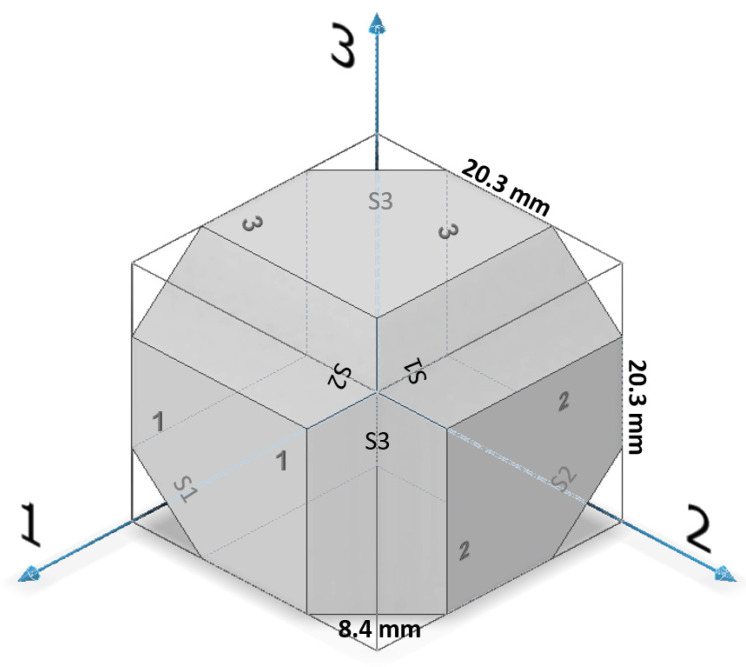
A polygon sample for the wrought and EBM Ti6Al4V alloys. Direction 3 is the rolling or build direction.

**Figure 2:**
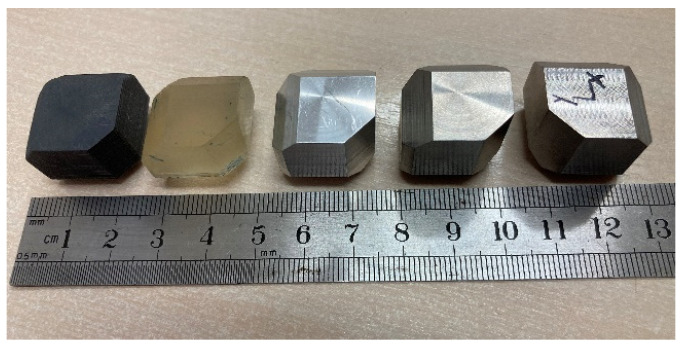
A CCD camera photo of five polygon samples—two fabricated from PLA polymer by the FDM AM technology (left), one made of commercial aluminum alloy by milling (center), and two made of Ti6Al4V—wrought (second from the right) and DED (right).

**Figure 3:**
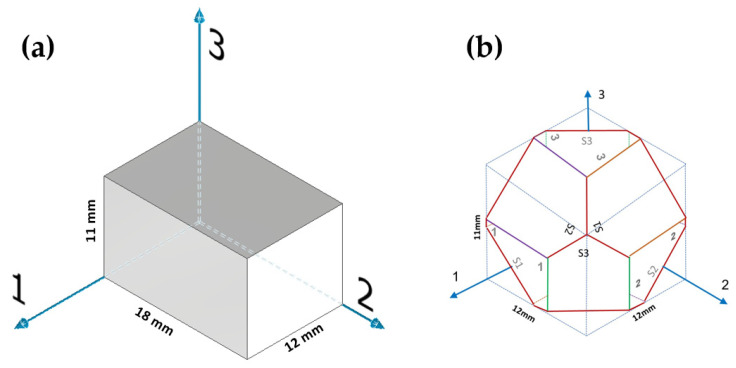
Two-step sample preparation and TOF measurement sequence for DED and SLM samples. (**a**) A 18 mm × 12 mm × 11 mm parallelogram sample utilized for measuring nine TOF *V*_ij_ (i,j = 1,2,3) values. (**b**) A small polygon sample utilized for measuring three TOF *V*_si_ (i = 1,2,3) values.

Both the wrought and the EBM samples for ultrasonic testing were milled to a polygon shape with dimensions of 20.3 mm × 20.3 mm × 20.3 mm, as shown in Figure 1. The polygon shape was fabricated according to the sample design presented in [44]. In order to achieve high accuracy (relative error below 0.3%), each wedge was parallel to its counterpart to a difference of less than 10 μm along the whole wedge and less than 4 μm in the measurement area (i.e., at the center of the wedge), and all wedge surfaces were polished. Figure 2 shows a CCD camera photo of five polygon samples—two made of AM Ti6Al4V (right), one made of by milling of a commercial aluminum alloy, and two made of polylactic acid (PLA) polymer AM by the fused deposition modeling (FDM) technology. The PLA and the aluminum samples were fabricated for feasibility tests, in order to verify the compatibility of the sample size and geometry with the ultrasonic transducers. The dimensions of the polygon were the largest possibly milled from the 1″ in diameter wrought rod and the EBM sample. In the case of the DED and SLM samples, the as-printed dimensions of the samples were too small to allow fabrication of a polygon sample in one-step milling, as the minimal sample diameter for the shear transducer is 0.25″. Therefore, sample preparation was utilized by a two-step milling process. First, a parallelogram with the dimensions of 18 mm × 12 mm × 11 mm was prepared (Figure 3a). Using this parallelogram sample, nine TOF *V*_ij_ (i,j = 1–3), three TOF longitudinal *V*_l,ii_ (i,j = 1–3), and six TOF shear *V*_ij_ (i,j = 1–3, i≠j) were measured. Next, the parallelograms were milled to a polygon with final dimensions of 12 mm × 12 mm × 11 mm (Figure 3b).

Using the so-obtained small polygon samples, three additional TOF *V*_si_ (i = 1–3) were measured.

**Figure 4:**
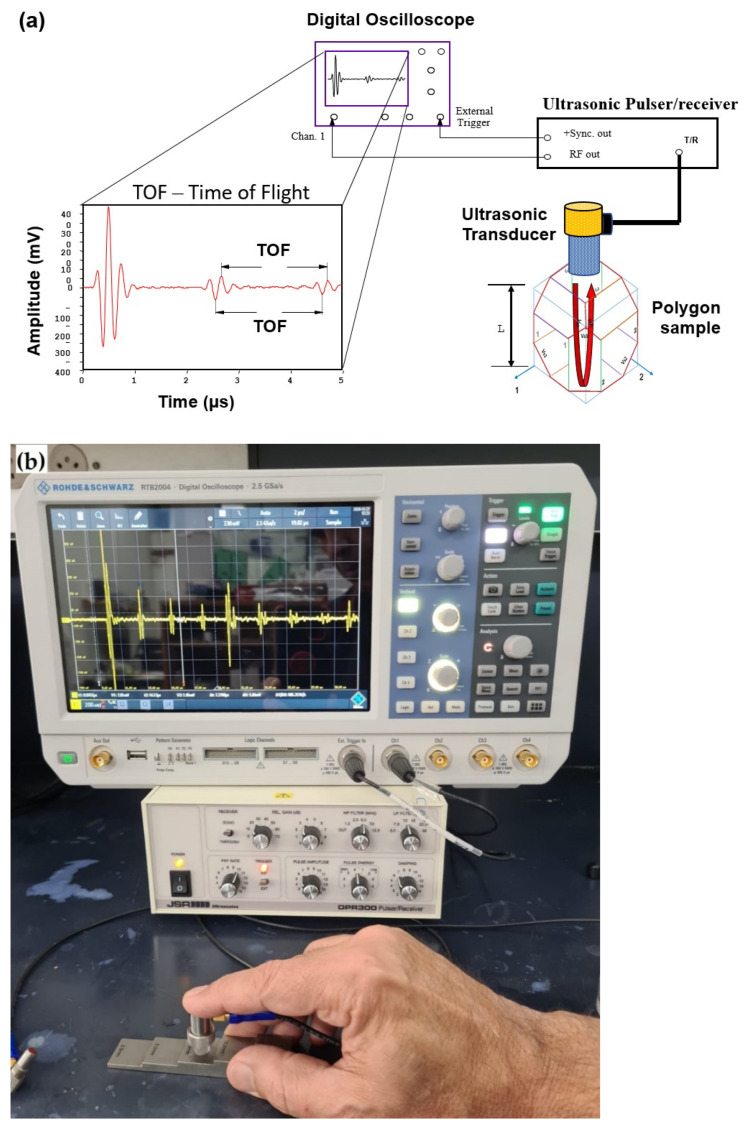
(**a**) Schematic illustration of the pulse-echo ultrasonic setup used for measuring the TOF in Ti6Al4V polygon samples. (**b**) Testing of a standard calibration specimen made of 304 stainless steel.

The distance between two parallel surfaces was determined with the aid of a micrometer. The SWVs and the TOF were measured using an ultrasonic pulse-echo setup with one ultrasonic transducer (Figure 4a). The setup consisted of a DPR300-M475-35 pulser/receiver (JSR Ultrasonics, Pittsford, NY, USA) and a digital RTB2004 oscilloscope (Rohde & Schwarz, Munich, Germany). The SWV was generated and sensed by the same acoustic probe (V203-RM, 10 MHz, 0.125”) for longitudinal waves, and a V156-RM probe (5 MHz, 0.25”) for shear waves, both from Olympus Czech Group, s.r.o. (Praha, Czechia). The oscillation direction of the shear waves is in line with the probe’s right-angle connector [34]. 

In the pulse-echo ultrasonic technique, an electric signal generated by a pulser is transformed to elastic wave by a piezoelectric probe. The waves traveling through the material are reflected from the back side and converted to electronic signals by the same piezoelectric probe. The SWV can be calculated from the measured time between two back-wall echoes (i.e., from the TOF) and the traveled distance between the two back-wall echoes, which is twice the sample thickness [34]. Material responses to the propagation of the waves include change in the attenuation coefficient, amplitude, and velocity of the ultrasonic wave, primarily due to scattering [25]. These responses are affected by both crystallographic orientation and defects, such as coherent and incoherent phase/grain boundaries, dislocations, vacancies, and process-induced defects, such as cracks and porosity [25].

The accuracy of the pulse-echo technique is exceptionally high [34,45] if the TOF is measured between the first and second echoes (reflection) from the sample’s back wall. This is because the parasitic TOF in the couplet between the ultrasonic transducer and the sample can be excluded (i.e., the TOF of the sound wave is the TOF of the sound wave moving forward and backward within the sample itself). When the sample is fully dense and has a coupling parallel face, the pulse-echo method is usually preferred over the through-transition (TT) method which employs two probes.

The accuracy of the pulse-echo system was evaluated with the aid of a 10 mm thick standard calibration specimen made of 304 stainless steel (see Figure 4b). A sound velocity of 5741 ± 4 m/s was thus measured, which is very close to the reported sound velocity of 5740 m/s in 302 stainless steel [46].

### 2.2. Density Measurements

The bulk relative density of the polygon samples was measured according to the Archimedes’ principle and ASTM B962–17 [47] using an ES 225SM-DR analytical balance with 0.01 mg readability and density analysis kit (Precisa Gravimetrica AG, Dietikon, Switzerland).

### 2.3. Elastic Constants Determination

The elastic behavior of orthotropic materials is defined by nine independent elastic constants. The well-established relationships between the ultrasonic phase velocities and the elastic constants (*C*_ij_) [48] enable determination of *C*_ij_ based on measurements of the longitudinal and shear velocities in several directions as well as the density [49]:

(7) $$C_{11}=\rho \times V{\;}_{11}^{2}$$

(8) $$C_{22}=\rho \times V{\;}_{22}^{2}$$

(9) $$C_{33}=\rho \times V{\;}_{33}^{2}$$

(10) $$C_{44}=\rho \times V{\;}_{23}^{2}\;=\;\rho \times V{\;}_{32}^{2}$$

(11) $$C_{55}=\rho \times V{\;}_{13}^{2}\;=\;\rho \times V{\;}_{31}^{2}$$

(12) $$C_{66}=\rho \times V{\;}_{12}^{2}\;=\;\rho \times V{\;}_{21}^{2}$$

(13) $$C_{12}=\sqrt{\left(C_{11}+C_{66}-2\rho \times V{\;}_{s3}^{2}\;\right)\times \left(C_{22}+C_{66}-2\rho \times V{\;}_{s3}^{2}\;\right)}-C_{66}$$

(14) $$C_{23}=\sqrt{\left(C_{22}+C_{44}-2\rho \times V{\;}_{s1}^{2}\;\right)\times \left(C_{33}+C_{44}-2\rho \times V{\;}_{s1}^{2}\;\right)}-C_{44}$$

(15) $$C_{13}=\sqrt{\left(C_{11}+C_{55}-2\rho \times V{\;}_{s2}^{2}\;\right)\times \left(C_{33}+C_{55}-2\rho \times V{\;}_{s2}^{2}\;\right)}-C_{55}$$

The directional Young’s moduli, shear moduli, and Poisson’s ratios (*ν*_ij_ (i,j = 1,2,3, i ≠ j)) can be derived from the elastic constants [42], as follows:

(16) $$E_{1}=\frac{D}{C_{22}C_{33}-C_{23}^{2}}$$

(17) $$E_{2}=\frac{D}{C_{11}C_{33}-C_{13}^{2}}$$

(18) $$E_{3}=\frac{D}{C_{11}C_{22}-C_{12}^{2}}$$

(19) $$G_{23}=G_{44}$$

(20) $$G_{13}=G_{55}$$

(21) $$G_{12}=G_{66}$$

(22) $$\nu_{23}=-\frac{E_{2}\left(C_{12}C_{13}-C_{23}C_{11}\right)}{D}$$

(23) $$\nu_{32}=-\frac{E_{3}\left(C_{12}C_{13}-C_{23}C_{11}\right)}{D}$$

(24) $$\nu_{13}=-\frac{E_{1}\left(C_{12}C_{23}-C_{13}C_{22}\right)}{D}$$

(25) $$\nu_{31}=-\frac{E_{3}\left(C_{12}C_{23}-C_{13}C_{22}\right)}{D}$$

(26) $$\nu_{12}=-\frac{E_{1}\left(C_{12}C_{23}-C_{12}C_{33}\right)}{D}$$

(27) $$\nu_{21}=-\frac{E_{2}\left(C_{12}C_{13}-C_{12}C_{33}\right)}{D}$$

(28) $$D=C_{11}C_{22}C_{33}-C_{11}C_{23}^{2}-C_{33}C_{12}^{2}-C_{22}C_{13}^{2}+2C_{12}C_{13}C_{23}$$

The Young’s moduli in all directions in space can be represented in radial coordinates, *E*(θ,φ), and be calculated as follows [50]:

(29) $$E\left(\theta ,\varphi \right)={\left(\begin{array}{l} \frac{\cos^{4}\theta }{E_{1}}+\frac{\sin^{4}\theta \times \cos^{4}\varphi }{E_{2}}+\frac{\sin^{4}\theta \times \sin^{4}\varphi }{E_{3}}+\cos^{2}\theta \times \sin^{2}\theta \times \cos^{2}\varphi I_{12}+\\ \sin^{4}\theta \times \sin^{2}\varphi \times \cos^{2}\varphi I_{23}+\sin^{2}\theta \times \cos^{2}\theta \times \sin^{2}\varphi I_{31} \end{array}\right)}^{-1}$$

where

(30) $$I_{\mathrm{ij}}=\frac{1}{G_{\mathrm{ij}}}-2\frac{\nu_{\mathrm{ij}}}{E_{\mathrm{ii}}}\;(i,j=1,2,3,\;i\neq j)$$

and φ and θ are the first rotation of the framework clockwise about the “1” axis and second rotation clockwise about the “3” axis (the new radial axis), respectively. In order to apply this method, 12 ultrasonic measurements must be conducted. 

### 2.4. Microstructure Characterization and Porosity Analysis

The Ti6Al4V polygon samples were analyzed by μCT for defects, including internal porosity. The samples to be scanned were placed on a glass and wax jig on top of the μCT 5-axis stage. For each sample, two scans were run, one with a region of interest (ROI) encompassing the entire sample and the second with the minimal voxel size possible, the sample size being the limiting factor. In the first scan, the sample was positioned to occupy the majority of the available area in the detector without having zones outside it. This position determines the effective magnification (and voxel size) in the scans. In the second scan, on the other hand, the limitation was to prevent the sample from hitting the X-ray source (thus, some zones on the sample were not scanned). A Phoenix v|tome|x m 240 system (Waygate Technologies, Wunstorf, Germany) with detail detectability of less than 1 µm was used for this purpose. The scanning parameters were: acceleration voltage of 180 kV, current of 40–100 µA, voxel size of 15–19 µm for the large samples and approximately 7.4 µm for the small close-up ROI scans, and a beam filter made of pure copper in varying thicknesses of 0.1–1.5 mm. A dynamic 41|200 large area detector with a timing setting between 333 and 1000 ms was used. During the scan, the sample was rotated 360°, and up to 3000 image positions per rotation were acquired, with three averages and one image skip at each angular position. 

A VGDefX algorithm (ver. 2.2) was used for the purpose of calculating the internal defects from the μCT data, namely, void and inclusion defects in the internal bulk analysis. Numerous challenges were encountered in the process of the μCT scan. The use of beam hardening correction (BHC) was necessary due to the mostly macro-homogeneous nature of the samples; the result was a good contrast between the main components of the sample—air and voids, Ti, and inclusions, where applicable. The large dataset and model from each scan posed a challenge for the VGDefX defect analysis algorithm, because the analysis of the full volume did not converge. To mitigate this problem, two solutions were used in parallel. First, an ROI analysis of the volume was used in key areas, such as visible defects. Second, a new volume was created, with binning the voxels effectively reducing the voxel count by a factor of eight, thus enabling the completion of the defect analysis for the samples. For each sample, selected analysis results were compared to make sure that no significant data was lost due to the binning of the volume. To further keep the defect analysis quick and relevant, the parameters were chosen to reflect the measurable size with the ultrasonic wavelength, and 1/4 of the smallest possible wavelength was chosen (200 μm). This decision kept the minuscule voids between the powder grains out and showed only true large voids. Thus, the results are comprised of the data analysis from the manual inspection of the non-binned volume and defect analysis of the binned volume. The μCT scans were conducted on the fractional volume of the samples. Two shapes were analyzed—#1 from the wrought alloy and EBM polygon and two parallelogram samples, #2 from the DED and SLM samples.

Microstructure characterization of the wrought rod and the EBM sample was performed to better explain the results of the ultrasonic pulse-echo analysis and polarity test. Both Ti6Al4V samples were sectioned into sections in two distinct directions corresponding to: (1) face 1, (2) face S1 (see Figure 1). The sectioned samples were ground using SiC grinding paper in the following sequence: (1) 320 grit, (2) 800 grit, (3) 1200 grit, (4) 2500 grit, and (5) 4000 grit, followed by polishing with 3 μm and 1 μm diamond suspensions. Fine polishing was made using 0.01 μm colloidal silica suspension. The microstructure characterization of the polished Ti6Al4V samples was done using SEM (Quanta 200 FEG, FEI, Waltham, MA, USA) equipped with an EBSD detector (NORDLYS II, Oxford Instruments, High Wycombe, UK), using an acceleration voltage of 20 kV and a step size of 0.15 μm. The data was processed using Aztec processing software. The EBSD analysis was conducted for the reconstruction of the α and β phases and for the analysis of grain size, aspect ratio, and crystallographic orientation.

## 3. Results and Discussion

### 3.1. Porosity and Its Effect on the Elastic Moduli

The porosity, *P*, in each sample was determined by Archimedes density measurements according to Section 2.2 and Equation (1), assuming a theoretical density *ρ*_t_ = 4.432 g/cm^3^ for Ti6Al4V [51]. The density and porosity values are tabulated in Table 2. It is evident that the porosity varied between 0.09 (DED) and 0.50% (SLM). It should be borne in mind that the values obtained from Archimedes density measurements are affected by the chemical composition of the printed alloy, element partitioning, surface roughness, open pores, the density of the starting powder, the evaporation of aluminum and gain of oxygen during the process, etc. Nevertheless, this method is the most common one for determining the density of AM materials, with some advantages over alternative techniques [9,37], and the one the international standards for PBF and DED of Ti6Al4V usually require. 

**Figure 5:**
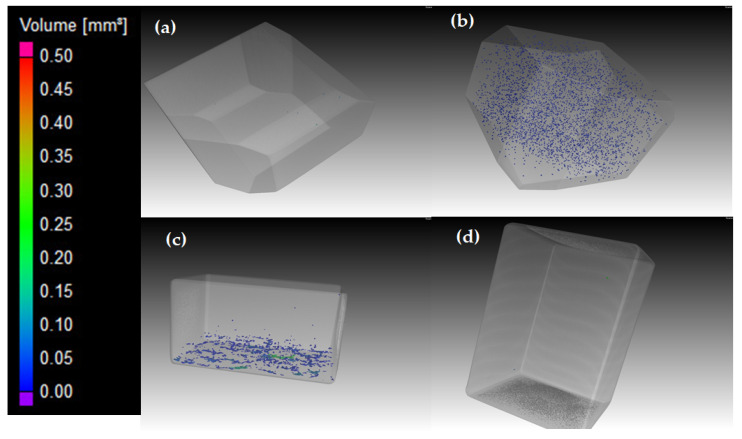
μCT 3D images of the Ti6Al4V samples. (**a**) Wrought polygon sample, (**b**) EBM polygon sample, (**c**) DED parallelogram sample, and (**d**) SLM parallelogram sample. The corresponding density values based on Archimedes measurements are given in Table 2.

To elucidate the effect of the pores’ size, shape, and distribution in the sample on the observed polarity and the measured elastic properties, μCT imaging of the samples was conducted. Three-dimensional μCT images of the four samples are shown in Figure 5. These images were analyzed by setting the probability threshold at 200 µm, i.e., only pores with a diameter larger than 200 µm are taken into account in the analysis.

Figure 5a reveals only few pores larger than 200 μm in diameter, in good agreement with the high measured (Archimedes) density of 99.80% in the wrought alloy. Figure 5b reveals many pores in the EBM alloy sample, the majority of which had volumes of about 0.01 mm^3^ (blue color), although the measured density of this sample (99.75%) was similar to that of the wrought alloy. The Archimedes density of the DED samples was the highest (99.90%), and the μCT image (Figure 5c) reveals that the pores are concentrated at the interface between the substrate plate and the build. Similar observations have been reported before for DED samples [7,37]. Most of the pores in this sample had volumes of ~0.01 mm^3^ (blue color), although some were larger (0.25 mm^3^, green color). The μCT image of the SLM sample (Figure 5d) reveals only few pores, although its Archimedes relative density was the lowest (99.50%). By lowering the probability threshold of the scan to 50 µm, significantly more (330) pores became apparent. The fact that many more pores were revealed by μCT in the EBM and DED samples than in the SLM sample, although the latter was less dense based on Archimedes measurements, is still puzzling. Some differences in the density values deduced from the Archimedes measurements vs. μCT have been reported before too [37]. However, such difference does not seem to explain the difference in the trends of EBM and DED vs. SLM in the current study. It should be borne in mind that different Ti6Al4V powders were used for the three AM processes, which differed in their size, oxygen, and trace element levels. μCT inclusion analyses of the DED and EBM samples revealed many evenly dispersed high-density defects, which may be related to iron contamination, which could originate from the powders (see chemical analyses results in Section 2.1). Such contamination could slightly increase the density in Archimedes measurements. 

### 3.2. Sound Wave Velocities and Elastic Constants Determination by the Dynamic Pulse-Echo Ultrasonic Testing

The SWVs were measured as described in Section 2.1. The SWVs measured on the four alloy samples are provided in Table 3. The maximum error in the longitudinal SWV value was 6 m/s, while that in the shear SWV value was 10 m/s. The elastic constants were calculated as explained in Section 2.3, Equations (7)–(15). The values of *C*_ij_ calculated for the four alloy samples are tabulated in Table 4. The maximum error in the calculated longitudinal elastic constant *C*_ii_ (i = 1,2,3) was 0.33 GPa, while that of the shear elastic constant *C*_jj_ (j = 4,5,6) was 0.28 GPa, and that of the shear elastic constants *C*_12_, *C*_13_, and *C*_23_ was 0.42 GPa.

The isotropic nature of the manufacturing process can be evaluated from the difference in the elastic constants of the same type, i.e., *C*_ii_ (i = 1,2,3), *C*_jj_ (j = 4,5,6), and *C*_12_, *C*_13_, and *C*_23_ reported in Table 4. It is apparent that these *C*_ij_ values were essentially the same for the three AM samples. The EBM sample exhibited perfect elastic isotropy, the DED sample showed lower values in the build direction (i.e., axis 3), while the SLM sample showed higher values in the build direction. In contrast, the commercial wrought Ti6Al4V alloy exhibited a highly anisotropic behavior.

The directional Young’s moduli, shear moduli, and Poisson’s ratios were derived from the elastic constants as explained in Section 2.3, Equations (16)–(27). The thus-derived values are tabulated in Table 5. The maximum error in Young’s modulus *E*_i_ (i = 1,2,3) was 0.51 GPa, that in the shear modulus *G*_ij_ was 0.28 GPa, and that in Poisson’s ratio was 0.005. For comparison, room-temperature values of the Poisson’s ratio and Young’s modulus that have been reported for Ti6Al4V are 0.33 and 106–146 GPa, respectively [52]. The highest value of the Young’s modulus was measured when the test direction was parallel to the high density of basal poles (α deformation texture). Ganor et al. [53] calculated Young’s moduli of 122.1 ± 1 and 108.0 ± 1 GPa for an EBM Ti6Al4V sample in the as-built condition and for commercial extruded rod-annealed sample, respectively. It should be noted that the EBM sample was built in the same machine under conditions similar to those used in the current study, and that the Young’s modulus values were calculated from only one stress–strain curve per material type. It should also be mentioned that measurement of the elastic constants by the dynamic ultrasonic technique has been claimed to be more accurate than determining them from tensile tests [9,35,37]. Nevertheless, both aforementioned comparisons support the reliability of the values reported herein in Table 5.

**Figure 6:**
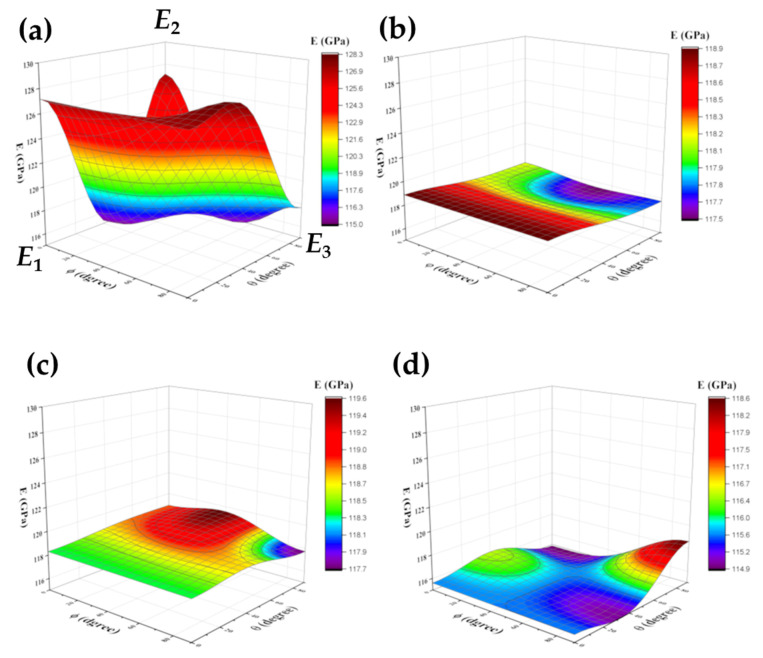
The 3D young’s modulus maps of Ti6Al4V samples fabricated by various methods: (**a**) commercial rod sample, (**b**) DED, (**c**) EBM, (**d**) SLM.

Figure 6 shows the four samples’ Young’s moduli variation as a function of the radial coordinates θ and φ (see Equations (29) and (30)). The 3D map of the wrought alloy sample is shown in Figure 6a; a significant anisotropy is clearly evident. Figure 6b,c show the 3D Young’s modulus maps of the DED and EBM samples, respectively. An almost flat behavior is evident, although the DED has a shallow maxima at (θ = 0°, φ = 0–90°), and the EBM has a shallow maxima at (θ = 90°, φ = 30°). The 3D Young’s modulus map of the SLM sample, Figure 6d, exhibits local maxima both at (θ = 30°, φ = 0°) and at (θ = 90°, φ = 90°). These 3D Young’s elastic modulus maps and the tensor of elastic constants are valuable for materials selection and finite element analyses in mechanical design. The fact that elastic properties depend on the manufacturing method and on the orientation relative to the rolling direction or the build direction should be taken into account.

Some discussion is provided here for the anisotropy in the elastic moduli and comparison between wrought and AM Ti6Al4V alloys. Pantawane et al. [54] measured the dynamic elastic constants of both SLM and wrought Ti6Al4V using the effective bulk modulus elastography (EBME) technique, and compared the results with the static elastic constants evaluated using the nanoindentation technique. The dynamic elastic constants were 5–8% lower than the static elastic constants. In addition, the static moduli of the SLM alloy were 22–26% lower than those of the wrought alloy. The density was 4.3925 ± 0.0835 and 4.493 ± 0.027 g/cm^3^ for the SLM and wrought alloys, respectively. The dynamic Young’s modulus, shear modulus, and Poisson’s ratio were 123 ± 3.11 GPa, 47.07 ± 1.31 GPa, and 0.3 ± 0.003 for the SLM alloy, and 138.06 ± 0.25 GPa, 53.43 ± 0.2 GPa, and 0.28 ± 0.0005 for the wrought alloy. In that work, however, all measurements were carried out on block cubes, and the elastic constants tensor was not derived. In a related work [25], the same group used an integrated EBME and shear wave velocity measurement approach and studied the texture driven elastic response of both SLM and wrought Ti6Al4V alloys. A change in the bulk elastic stiffness at shear wave planes oriented at 45° and 90° with respect to the plane normal to the build direction was observed due to a drop in the shear SWV at these orientations. EBSD analysis was used to relate this difference to the orientations of α′ crystallographic variants within prior columnar β grains, which increased the probability of aligning the soft directions to the shear vibration direction. Borovkov et al. [55] developed an elastic-plastic model for EBM Ti6Al4V. The model considered three Young’s moduli, three shear moduli, and three Poisson’s ratios as elastic properties, and six coefficients describing the Hill yield criterion. Measurements were also conducted by uniaxial tension and torsion tests. The Young’s moduli, shear moduli, and Poisson’s ratios measured along three axes were 121.9–124.2 GPa, 37.5–42.0 GPa, and 0.25–0.26, respectively. Only slight anisotropy was recognized along three perpendicular directions.

The level of the porosity, the pores’ sizes and shapes, and the distribution of pores in the matrix influence the elastic moduli and isotopy. μCT analysis of the wrought polygon revealed a pore-free material, therefore the anisotropy in the elastic modulus of the wrought alloy cannot be related to porosity, but to the microstructure or stress distribution in the sample. μCT analysis of the SLM sample, on the other hand, revealed high number of very small pores, evenly distributed in the matrix, with a total of 0.12% porosity. The EBM sample had similar characteristics; it contained a high number of medium-size pores, evenly distributed, and a total of 0.21% porosity. Evenly distributed pores can lead to a uniform decrease of the elastic modulus. This μCT analysis is in good agreement with the 3D Young’s modulus maps (Figure 6c,d, respectively) and with the fact that *C*_11_ ≅ *C*_22_ ≈ *C*_33_ of the SLM and EBM samples. μCT analysis of the DED sample revealed substantial amount of pores at the bottom of the sample, and a total porosity of 0.19%. This is in good agreement with the fact that *C*_33_ of DED sample is more than 1% lower than *C*_11_ and *C*_22_. This anisotropy can be related to the local porosity at the bottom of the sample. 

### 3.3. The Effect of Polarization Orientation on the Ultrasonic Waveform

From Table 3 it is evident that the polarity in any direction in the EBM sample is negligible, i.e., *V*_12_ ≅ *V*_13_, *V*_21_ ≅ *V*_23_, and *V*_31_ ≅ *V*_32_. In contrast, polarity is evident in the *x* and *y* directions of the wrought alloy, namely, *V*_12_ ≠ *V*_13_ and *V*_21_ ≠ *V*_23_, but not in the *z* direction, i.e., *V*_31_ is almost the same as *V*_32_.

**Figure 7:**
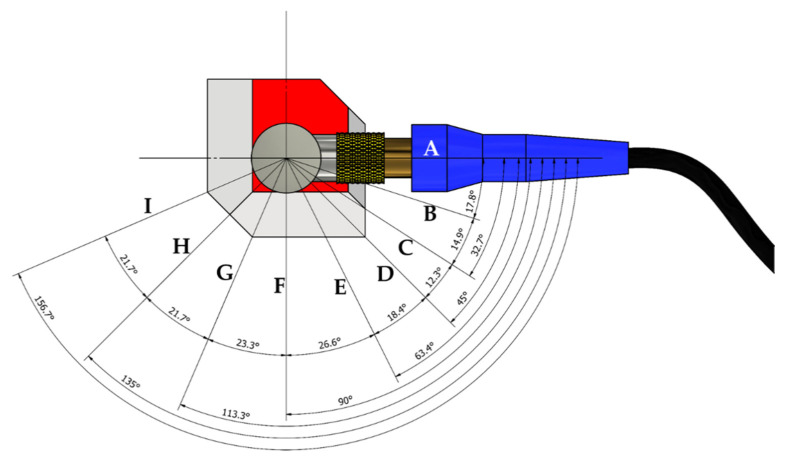
Schematic setup for measuring the shear SWV at different angles. A–I represent different angles of the acoustic probe at which the waveforms were captured.

In order to elucidate the influence of the orthotropic nature on the wrought alloy and on the EBM polygon sample, the polarity was determined by calculating the relative SWV dissimilarity while the angle of the acoustic probe was changed. Figure 7 shows the schematic setup for the polarity test. Polarity tests were conducted on the EBM and wrought samples. The probe was attached to the *x* face (axis 1) of the polygon, and each of the waveforms was captured at different angles (A–I). For example, when the probe was attached to the *x* face of the polygon sample in a position where the cable connector is pointing to the A direction, the oscillation was in the *y*-direction (i.e., *V*_12_ was measured). When the connector pointed to the F direction, the wave oscillated in the *z*-direction (i.e., *V*_13_). The *z*-direction was defined as the build or rolling direction in the EBM and in the wrought samples, respectively. 

**Figure 8:**
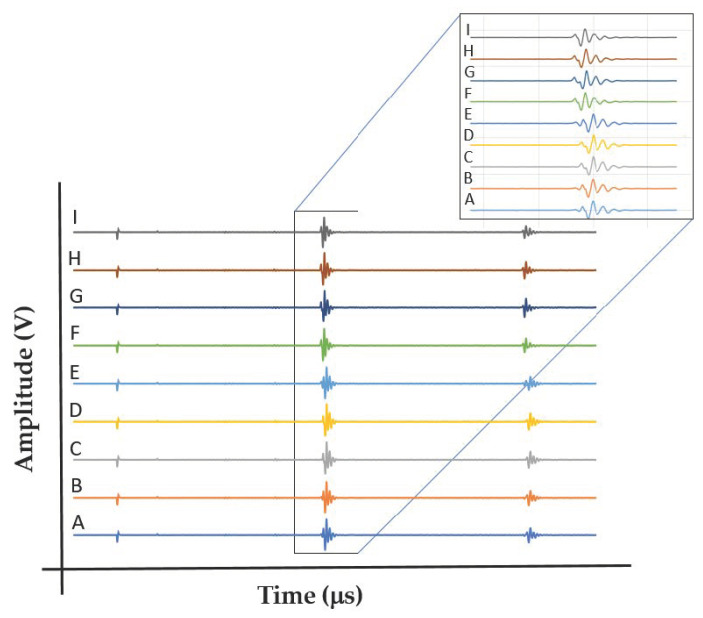
Nine shear SWVs at different angles (A–I) in the EBM sample. Zoom-in on the first echo reveals a small shit in the TOF.

Figure 8 shows nine shear waves that were amplified by the receiver in a polarity test conducted on the EBM polygon sample, and were then captured and saved with the aid of the digital oscilloscope. On the left side of the waveform, the first small signal is the reflection of the generating pulse from the surface of the sample, while the other two waveforms are the first and the second echoes from the back wall of the sample. In the upper waves we can see in detail the first echo. There is a small shift in the TOF between these nine waves. The small shift of the signal at position A represents the *V*_12_ wave, while position F represents the *V*_13_ wave. A small polarity is evident in the EBM sample. This correlates well with the SWV in the EBM sample, as reported in Table 3 (*V*_12_ vs. *V*_13_). 

**Figure 9:**
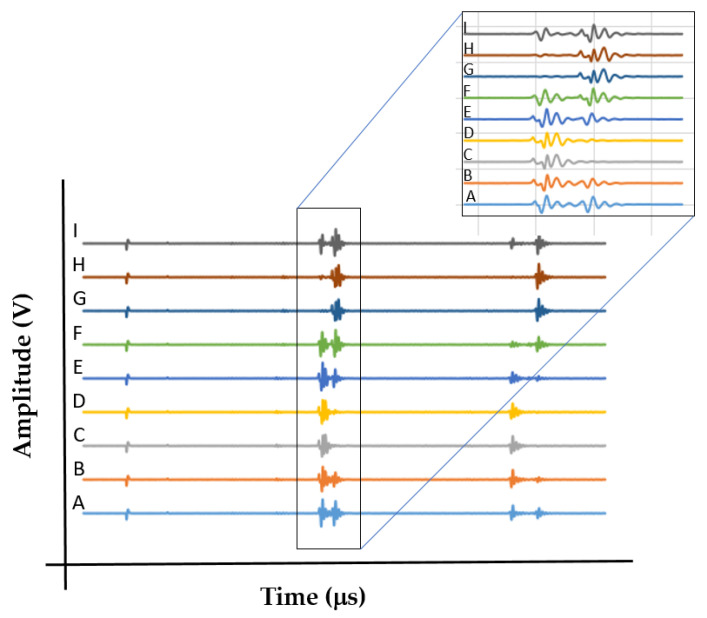
Nine shear sound waves at different angles (A–I) in the wrought alloy sample. The first and second echoes are evident. The zoom-in reveals the appearance and disappearance of the first and second reflection waves.

The same polarity test using the same setup was conducted on the wrought polygon sample. Figures S1 and S2 (Supplementary Materials) illustrate the probe position on the wrought alloy polygon sample in directions A/F and C/G, respectively. Figure 9 shows the nine shear waveforms in this case. A significant change in the waveform shape as a function of the angle of oscillation (A–I) is evident. In addition, the SWVs change significantly as a function of orientation. For example, in the A direction *V*_12_ = 3.429 km/s, whereas in the F direction *V*_13_ = 3.118 m/s. This implies ~10% increase in the shear SWV, which is considered to be a substantial polarity effect. The most puzzling measurements were waveforms C and G, in which only one echo was observed.

### 3.4. Microstructure Characterization

**Figure 10:**
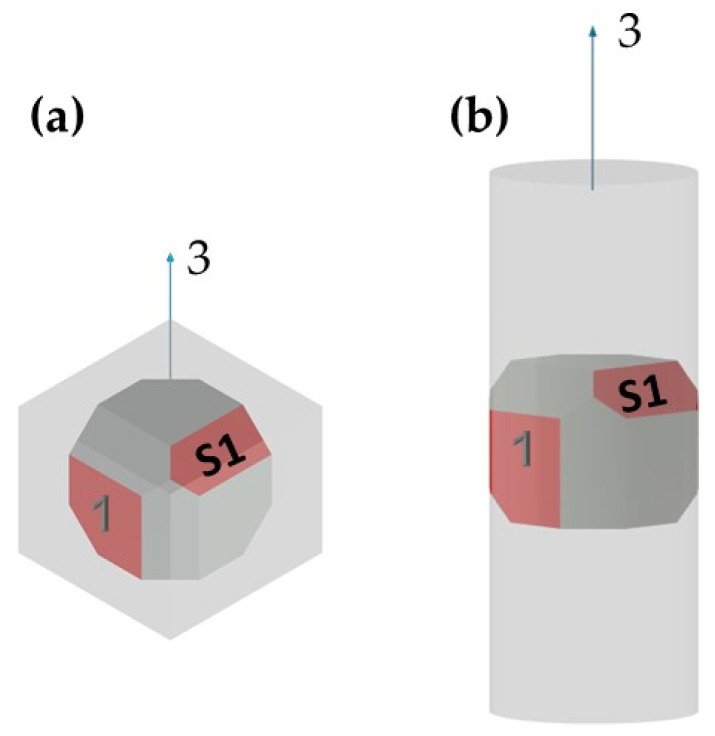
Illustration of the polygon DED (**a**) and wrought (**b**) samples. The red facets 1 and S1 were analyzed by SEM-EBSD.

Mechanical properties anisotropy in materials due to microstructure anisotropy is a well-known paradigm [56], although the correlation between elastic properties anisotropy and microstructure anisotropy in Ti6Al4V remains unclear. This calls for fundamental characterization of the microstructures of the Ti6Al4V alloys studied herein. EBSD analyses were carried out on both the wrought and EBM Ti6Al4V samples, determining the grain size, grain morphology, and crystallographic orientation and phases. Each of the two samples was analyzed in two distinct directions corresponding to facets 1 and S1 (Figure 10). In both samples, facet 1 is the face to which the ultrasonic probe was attached in the polarity test, whereas facet S1 is the direction in which the double wave appeared in the wrought alloy sample.

**Figure 11:**
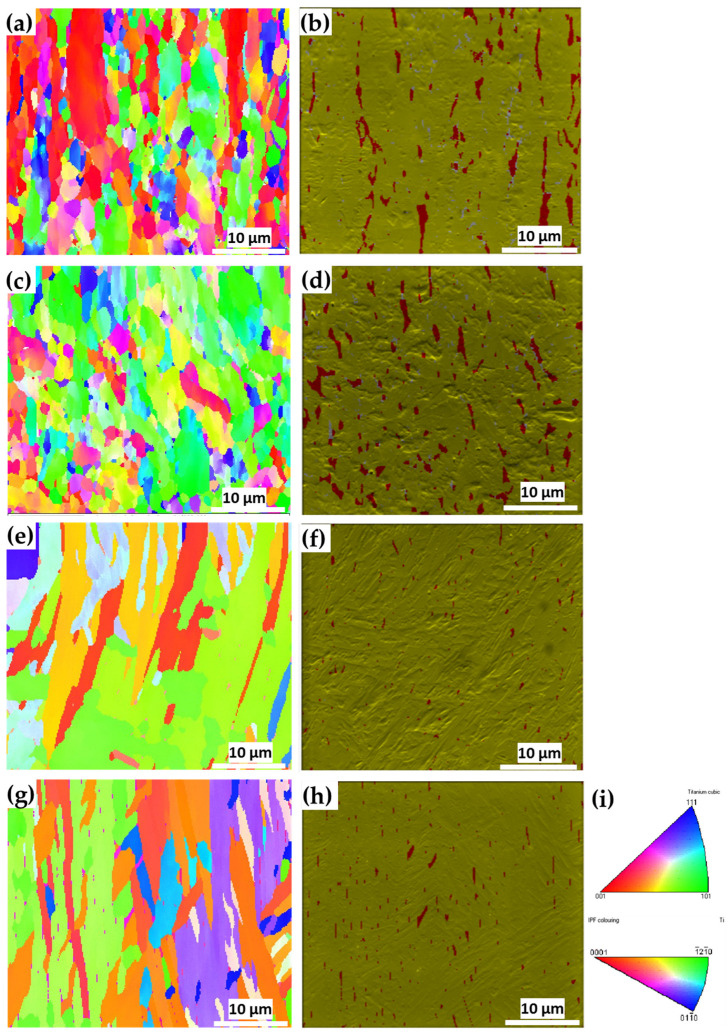
EBSD *z*-direction IPF maps and SEM SE images with phase identification overlay (α phase in green-yellow coloring, β phase in red coloring) for (**a**,**b**) wrought Ti6Al4V sample, facet 1; (**c**,**d**) wrought Ti6Al4V sample, facet S1; (**e**,**f**) EBM Ti6Al4V sample, facet 1; (**g**,**h**) EBM Ti6Al4V sample, facet S1; (**i**) IPF coloring schemes correspond to the β-phase (top) and α-phase (bottom).

**Figure 12:**
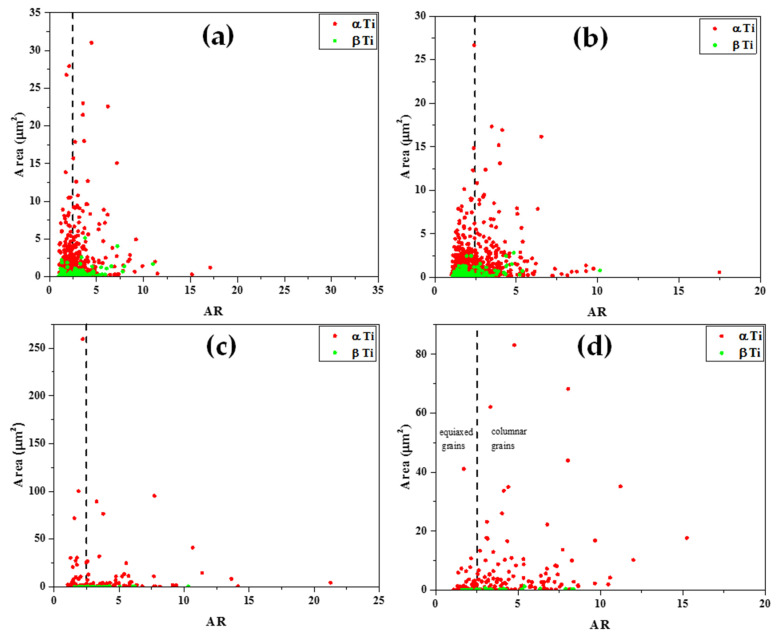
Grain area vs. aspect ratio (AR) plots of (**a**) wrought Ti6Al4V sample, facet 1; (**b**) wrought Ti6Al4V sample, facet S1; (**c**) EBM Ti6Al4V sample, facet 1; (**d**) EBM Ti6Al4V sample, facet S1.

Figure 11a,c shows the *z*-direction inverse pole figure (IPF) maps for the wrought Ti6Al4V sample in the two analyzed directions. The presented maps are comprised of both equiaxed and columnar grains. For both the wrought and EBM samples, no obvious columnar to equiaxed transition layers were observed (Figure 11). Figure 11b,d shows the corresponding SEM secondary electron (SE) images combined with phase identification overlay for the wrought Ti6Al4V samples in both analyzed directions (facet 1 and S1). The morphology of the β-phase grains corresponding to facet 1 of the wrought sample is more elongated than that in facet S1 sample. This matches the elongated grain structure along the rolling direction in Figure 11a. The microstructure of facet S1 shows less elongated grains as it is a cross-section cut at 45° relative to facet 1. The EBSD phase identification analysis reveals that the microstructure of the wrought alloy is composed of ~94% α-phase and ~6% β-phase (Table S1, Supplementary file). This is in good agreement with a recent study [38] that reported 6.13% β-phase in this wrought alloy based on X-ray diffraction (XRD) analysis. Figure 11e,g shows the *z*-direction IPF maps of the EBM Ti6Al4V samples in the two analyzed directions. These IPF maps are comprised of both equiaxed and columnar grains, which are quantitatively analyzed in Figure 12. 

Compared with the grain size of the wrought alloy sample, the microstructure of the EBM sample is comprised of much larger grains, and thus a lower number of grains within the same field of view. Furthermore, it can be seen that the β-phase grains in the EBM sample exhibit a fine precipitate-like morphology. This is in good agreement with a recent study that investigated the phase evolution of Ti6Al4V alloy fabricated using the EBM process [57]. The EBSD phase identification analysis reveals that the microstructure of the EBM sample along the build direction is composed of ~99% α-phase and ~1% β-phase (Table S1, Supplementary file). This is in good agreement with recent reports on ~1% β-phase in EMB-fabricated Ti6Al4V alloy, based on EBSD analysis [53,57]. 

The grain area as a function of the aspect ratio (AR) of both β and α phases was determined for each individual grain and for each sample, as shown in Figure 12. For all samples, only grains with an area above 0.11 μm^2^ were considered for the purpose of this analysis, in order to emphasize larger grains whose interaction with the propagating sound wave (at 5 MHz) is substantially higher in comparison to smaller grains. Here, AR > 2.5 represents columnar grains, whereas AR < 2.5 represents equiaxed grains [58].

**Figure 13:**
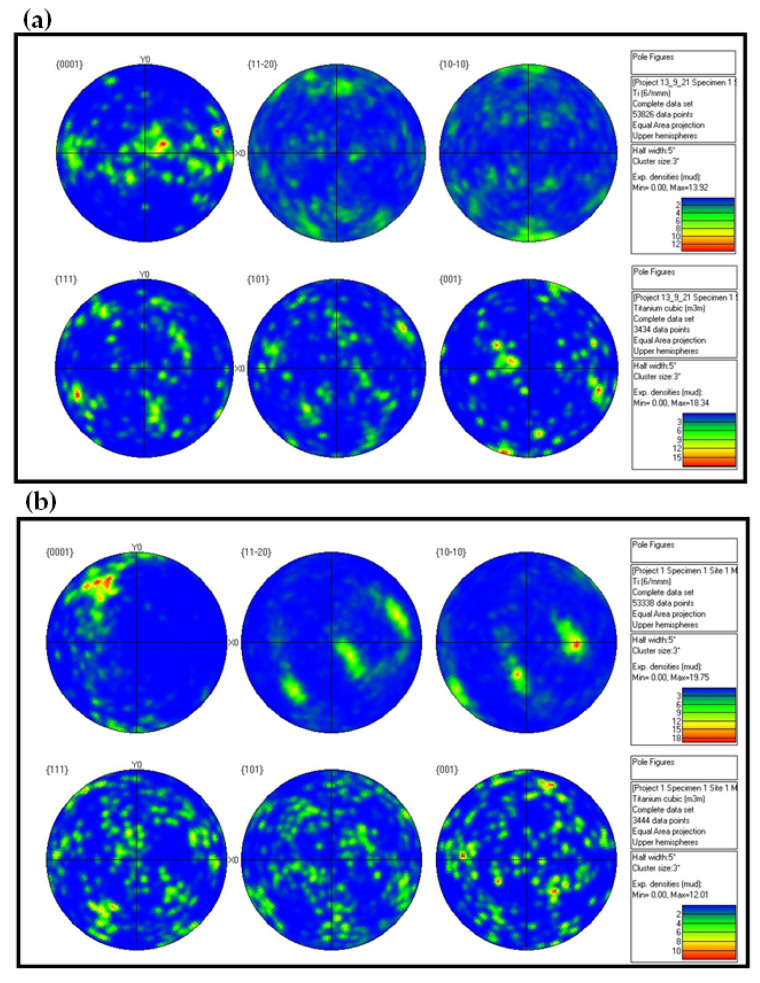

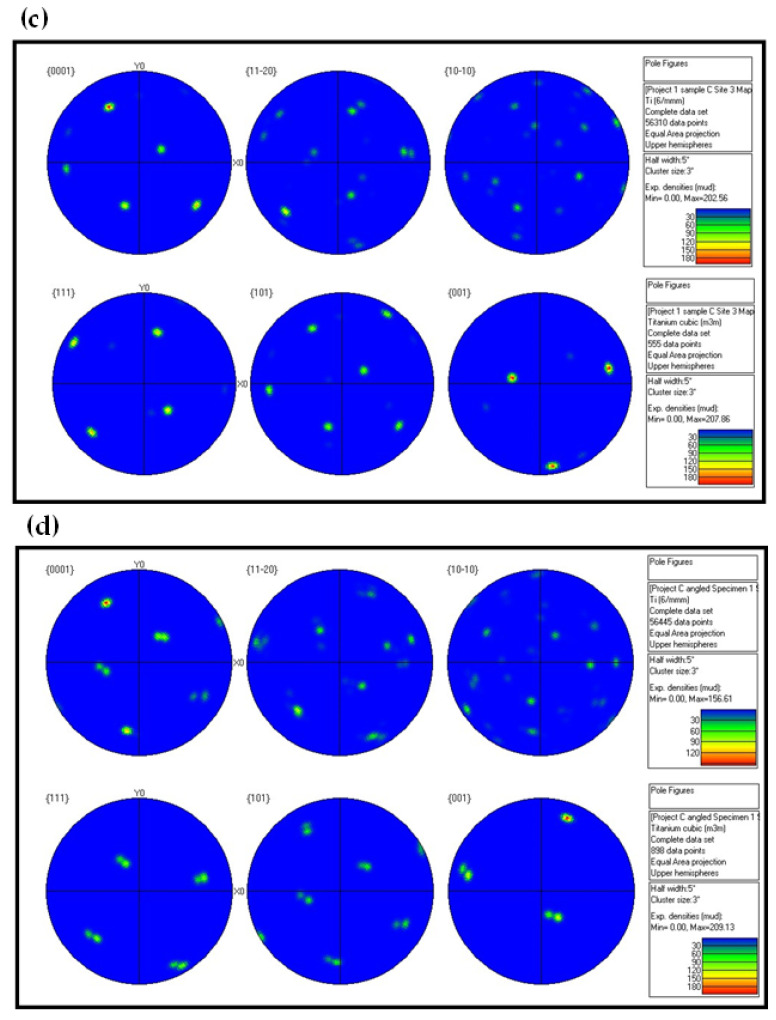
Pole figures corresponding to α-phase (top) in principle orientations {0001}, {01-10}, and {-12-10}, and β (bottom) in principle orientations {111}, {101}, and {001}, for: (**a**) wrought Ti6Al4V sample, facet 1; (**b**) wrought Ti6Al4V sample, facet S1; (**c**) EBM Ti6Al4V sample, facet 1; (**d**) EBM Ti6Al4V sample, facet S1.

Two main ultrasonic anisotropies were revealed in the polarity test. The first one is based on the fact that in both hot-rolled (wrought) and AM alloys, elongated grains typically form along the rolling or build direction (axis 3 in Figure 10). Elongated grains in different crystallographic orientations may cause different shear SWVs in different polarities. Another possible explanation refers to the presence of large, elongated β-phase along the α-phase grain boundaries. Figure 11 and Figure 12 show that the wrought alloy sample exhibits smaller elongated grains than the EBM alloy sample while also containing an increased concentration of large, elongated β-phase. The presence of large, elongated β-phase in the wrought alloy sample can serve as the root cause of the observed difference in the shear SWVs and the observed double-hump waveform. Furthermore, neither of the two alloys exhibited preferred crystal orientation of the elongated grains (Figure 13). Therefore, the first aforementioned explanation may be ruled out, while the second explanation is very probable. Based on the EBSD analysis, there is ~6% β-phase in the wrought alloy and only ~1% β-phase in the EBM alloy (Table S1, Supplementary file). Furthermore, from Figure 11b,d it is clear that the β-phase in the wrought alloy forms as a thin layer along grain boundaries. The shear SWV is affected by this β-phase layer, depending on the waveform polarity. Different SWVs, either perpendicular or parallel to the β-phase layer, are responsible for the exact 90° between the fast-mode direction C and the slow-mode direction G (see Figure 9 and Figure S2).

## 4. Summary and Conclusions

The dynamic pulse-echo ultrasonic technique and specially designed polygon samples were employed to measure 12 sound wave velocities (SWVs) in Ti6Al4V fabricated by four different processes: hot rolling (wrought), electron beam melting (EBM) powder-bed fusion (PBF), selective laser melting (SLM) PBF, and Laser Engineered Net Shaping (LENS) directed energy deposition (DED). In conjunction with Archimedes density measurements, these SWVs were used to derive the tensor of elastic constants (*C*_ij_), elastic moduli (including the Young’s modulus, shear modulus, and Poisson’s ratio), and the three-dimensional Young’s moduli maps. Electron backscatter diffraction (EBSD) and micro-computed tomography (μCT) were employed to characterize the grain size and orientation as well as porosity and other defects which could explain the difference in the measured elastic constants of the four materials. 

Although the three AM samples differed in the level of porosity, they all exhibited similar values of elastic constants and essentially elastic isotropy. Three-dimensional Young’s modulus further supported this and illustrated the high anisotropy in the elastic constants of the wrought alloy. EBSD analysis revealed that the EBM alloy contained larger and more elongated grains than the wrought alloy, yet the latter exhibited substantial polarity. The β-phase content in the wrought alloy was significantly higher than in the EBM alloy (~6% vs. ~1%, respectively). The existence of the β-phase in the wrought alloy as a thin layer along grain boundaries could cause the shear acoustic wave in the wrought alloy to split when the atoms oscillate either perpendicular or parallel to the β-phase layer. It cannot be excluded that the differences in the elastic constants over different samples might be caused, among others, by small differences in chemical composition (including oxygen), as evident from Section 2.1. The results of this study indicate that the elastic properties of AM Ti6Al4V vary only slightly in different directions. Furthermore, since the texture of the analyzed AM samples is negligible, the presence of large columnar grains probably remains the main factor negatively affecting the tensile strength and fatigue properties. 

The finding that the elastic properties depend on the manufacturing process and on the angle relative to either the rolling direction or the AM build direction should be taken into account in the design of products. The data reported herein is valuable for materials selection and finite element analyses in mechanical design. The pulse-echo measurement procedure employed in this study may be further adapted and used as a non-destructive testing (NDT) technique for quality control of AM materials and parts. The ultrasonic velocity may be utilized to determine the exact building angle of the finished part without the necessity to either rely on information provided by part manufacturer or conduct destructive tests.

## Figures and Tables

**Table 1:** Twelve sound wave velocities measured using the polygon method.

No.	Velocities Notation	Wave Type	Propagation Direction	Polarization Direction	Visual Description
1	*V*_11_	Longitudinal	1		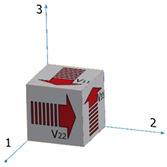
2	*V*_22_	Longitudinal	2		
3	*V*_33_	Longitudinal	3		
4	*V*_12_	Shear	1	2	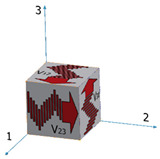
5	*V*_23_	Shear	2	3	
6	*V*_31_	Shear	3	1	
7	*V*_13_	Shear	1	3	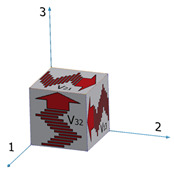
8	*V*_32_	Shear	3	2	
9	*V*_21_	Shear	2	1	
10	*V*_s1_	Shear	23	1	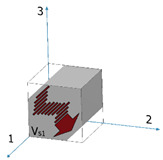
11	*V*_s2_	Shear	13	2	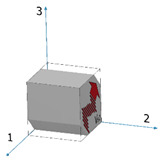
12	*V*_s3_	Shear	12	3	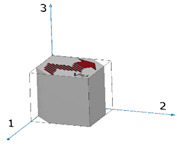

**Table 2:** The analyzed density and the porosity of the commercial Ti6Al4V rod sample and in the three Ti6Al4V AM samples.

Property	Wrought	EBM	DED	SLM
Density, *ρ* (g/cm^3^)	4.423 ± 0.0005	4.421 ± 0.0005	4.428 ± 0.0008	4.410 ± 0.0008
Relative density (%)	99.80	99.75	99.91	99.50
Porosity (%)	0.20	0.25	0.09	0.50

**Table 3:** Sound wave velocities (SWVs) in the commercial wrought Ti6Al4V alloy sample and in the three AM Ti6Al4V samples (*n* = 6). The maximum error (calculated based on the standard deviation and instrumental error) in the longitudinal SWV is 6 m/s, the maximum error in the shear SWV is 10 m/s.

No.	Velocities Notation	Wrought Alloy (km/s)	EBM (km/s)	DED (km/s)	SLM (km/s)
1	*V*_11_	6.258	6.191	6.172	6.161
2	*V*_22_	6.258	6.202	6.165	6.170
3	*V*_33_	6.099	6.204	6.136	6.193
4	*V*_23_	3.110	3.201	3.179	3.147
5	*V*_32_	3.096	3.200	3.174	3.145
6	*V*_13_	3.118	3.203	3.180	3.145
7	*V*_31_	3.098	3.202	3.160	3.146
8	*V*_12_	3.329	3.171	3.197	3.155
9	*V*_21_	3.330	3.199	3.176	3.143
10	*V*_s1_	3.236	3.178	3.178	3.167
11	*V*_s2_	3.238	3.172	3.190	3.188
12	*V*_s3_	3.350	3.198	3.182	3.121

**Table 4:** The elastic constants, *C*_ij_, of the commercial wrought Ti6Al4V alloy sample and the three AM Ti6Al4V samples (*n* = 6). The maximum error (calculated based on the standard deviation and instrumental error) in the longitudinal elastic constants *C*_ii_ (i = 1,2,3) is 0.33 GPa, the maximum error in the shear elastic constants *C*_jj_ (j = 4,5,6) is 0.28 GPa, the maximum error in the shear elastic constants *C*_23_, *C*_13_, and *C*_12_ is 0.42 GPa.

No.	Elastic Constant	Wrought Alloy (GPa)	EBM (GPa)	DED (GPa)	SLM (GPa)
1	*C*_11_	173.2	169.4	168.7	167.4
2	*C*_22_	173.2	170.0	168.3	167.9
3	*C*_33_	164.5	170.2	166.7	169.1
4	*C*_44_	42.6	45.3	44.7	43.6
5	*C*_55_	42.7	45.3	44.5	43.6
6	*C*_66_	49.0	44.9	45.0	43.7
7	*C*_23_	76.2	80.8	78.0	80.1
8	*C*_13_	76.1	80.8	77.6	78.6
9	*C*_12_	74.0	79.3	78.8	81.7

**Table 5:** The elastic moduli, *E*_ij_, of the commercial wrought Ti6Al4V alloy sample and the three AM Ti6Al4V samples (*n* = 6). The maximum error (calculated based on the standard deviation and instrumental error) in Young’s moduli *E*_i_ (i = 1,2,3) is 0.51 GPa, the maximum error in the shear moduli *G*_ij_ (i,j = 1,2,3, i ≠ j, *G*_ij_ = *G*_ji_) is 0.28 GPa, the maximum error in Poisson’s ratio *ν*_ij_ (i,j = 1,2,3, i ≠ j) is 0.005.

No.	Elastic Modulus	Wrought Alloy (GPa)	EBM (GPa)	DED (GPa)	SLM (GPa)
1	*E*_1_	127.17	118.30	118.88	115.60
2	*E*_2_	127.10	118.88	118.16	114.86
3	*E*_3_	117.68	117.74	117.76	118.62
4	*G*_23_	42.58	45.28	44.69	43.65
5	*G*_13_	42.73	45.33	44.51	43.63
6	*G*_12_	49.03	44.85	44.97	43.73
7	*ν*_23_	0.335	0.333	0.326	0.315
8	*ν*_32_	0.306	0.308	0.323	0.326
9	*ν*_13_	0.348	0.332	0.327	0.303
10	*ν*_31_	0.320	0.307	0.326	0.311
11	*ν*_12_	0.280	0.281	0.311	0.342
12	*ν*_21_	0.282	0.281	0.313	0.340

## Data Availability

All relevant data is contained within this article and the Supplementary Materials.

## Supplementary Materials

The following are available online at https://www.mdpi.com/article/10.3390/ma15020638/s1. Table S1: Phase contents in the wrought and in the EBM Ti6Al4V samples, as determined using electron backscatter diffraction (EBSD). Figure S1: Illustration of the polygon sample of the wrought alloy at directions A (a) and F (b). The corresponding sound wave velocity notations are *V*_12_ and *V*_13_, respectively. Figure S2: Illustration of the polygon sample of the wrought alloy at directions C (a) and G (b). The corresponding sound wave velocity notations are *V*_1C_ and *V*_1G_, respectively.
