# Supplementary material for: Measurement of the Anisotropic Dynamic Elastic Constants of Additive Manufactured and Wrought Ti6Al4V Alloys
Source: Materials (Basel). 2022 Jan 15;15(2):638. doi: 10.3390/ma15020638 (PMC8779917; doi:10.3390/ma15020638)
Supplement: Supplementary file 1 [file materials-15-00638-s001.zip › materials-1541183-supplementary.pdf]

materials

Supplementary

Measurement of the Anisotropic Dynamic Elastic Constants of Additive Manufactured and Wrought Ti6Al4V Alloys

Table S1. Phase contents in the wrought and in the EBM'ed Ti6Al4V samples, as determined using electron backscatter diffraction (EBSD).

Material, Facet.	Phase	Phase Fraction (%)
Wrought, facet 1	α (hcp)	94.27
	β (bcc)	5.73
Wrought, facet S1	α (hcp)	94.22
	β (bcc)	5.78
EBM, facet 1	α (hcp)	98.91
	β (bcc)	1.08
EBM, facet S1	α (hcp)	98.26
	β (bcc)	1.74

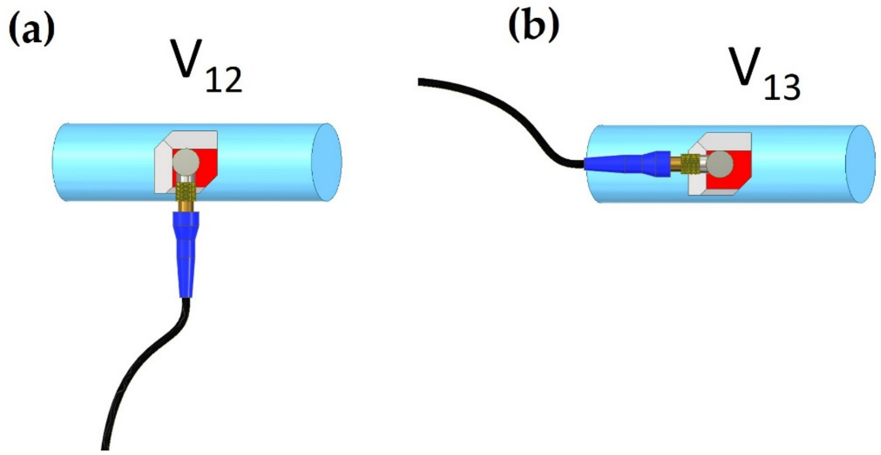

Figure S1. Illustration of the polygon sample of the wrought alloy at directions A (**a**) and F (**b**). The corresponding sound wave velocity notations are V_{12} and V_{13}, respectively.

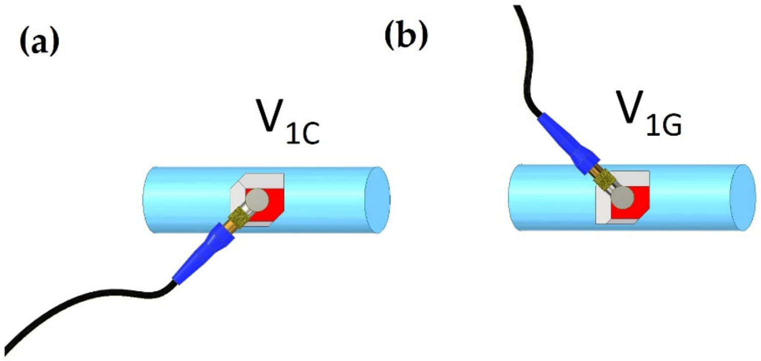

Figure S2. Illustration of the polygon sample of the wrought alloy at directions C (**a**) and G (**b**). The corresponding sound wave velocity notations are V_{1C} and V_{1G}, respectively.
